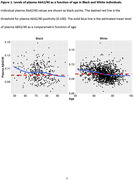# Age of plasma Aβ42/40 positivity in Black and White individuals

**DOI:** 10.1002/alz70856_106922

**Published:** 2026-01-08

**Authors:** Chengjie Xiong, Jingqin Luo, David A. Wolk, Leslie M. Shaw, Erik D Roberson, Randall J. Bateman, John C. Morris, Suzanne E. Schindler

**Affiliations:** ^1^ The Charles F. and Joanne Knight Alzheimer Disease Research Center, St. Louis, MO, USA; ^2^ Department of Surgery, Washington University, St. Louis, MO, USA; ^3^ University of Pennsylvania, Philadelphia, PA, USA; ^4^ Perelman School of Medicine, University of Pennsylvania, Philadelphia, PA, USA; ^5^ University of Alabama at Birmingham, Birmingham, AL, USA; ^6^ Washington University, Saint Louis, MO, USA; ^7^ Knight Alzheimer Disease Research Center, Washington University School of Medicine, St. Louis, MO, USA

## Abstract

**Background:**

Whereas longitudinal data are needed to pinpoint the exact age when individuals become positive for biomarkers of Alzheimer disease (AD), cross‐sectional data can be used to examine the typical age of biomarker positivity across groups. Using cross‐sectional data, we estimated the age when groups of self‐identified Black and White individuals reached a threshold for plasma Aβ42/40 positivity.

**Methods:**

We assembled plasma samples and data from a large cohort of 324 Black or African American and 1,547 White individuals from three AD Research Centers (Washington University, University of Pennsylvania, and University of Alabama at Birmingham). Plasma Aβ42/40 was measured with C2N Diagnostics mass spectrometry‐based assays. Locally estimated scatterplot smoothing (LOESS) was used to estimate the mean levels of plasma Aβ42/40 as a nonparametric function of age. Statistical calibration was then used to estimate the age when plasma Aβ42/40 reached the threshold for positivity (0.100). Analyses were performed with and without matching the Black and White groups by major AD covariates, and also in groups with and without comorbidities.

**Results:**

Unmatched analyses revealed an estimated age at plasma Aβ42/40 positivity of 69.6 years for the group of White participants and 86.9 years for the group of Black participants. Black participants (*n* = 317) were then matched 1:2 with White participants (*n* = 634) by age, sex, APOE ε4 carrier status, global Clinical Dementia Rating (CDR) (CDR=0, 0.5, >=1), and years of education (>12 years vs. <=12 years). The matched analyses estimated an age at plasma Aβ42/40 positivity of 68.4 years for the group of White participants and 86.9 years for the group of Black participants. Interestingly, participants with hypertension, stroke, or diabetes had a later age at plasma Aβ42/40 positivity.

**Conclusions:**

The typical age at plasma Ab42/40 positivity may depend on racialized group and also medical comorbidities. These results are consistent with recent reports that groups of Black individuals have a lower incidence of AD biomarker positivity compared to groups of White individuals. These findings may aid design and analyses of future clinical trials of AD.